# Exploring Nanofiltration for Transport of Small Molecular Species for Application in Artificial Kidney Devices to Treat End-Stage Kidney Disease

**DOI:** 10.3390/membranes15060168

**Published:** 2025-06-02

**Authors:** Haley Duncan, Christopher Newton, Jamie Hestekin, Christa Hestekin, Ira Kurtz

**Affiliations:** 1 Ralph E. Martin Department of Chemical Engineering, University of Arkansas, Fayetteville, AR 72701, USA; hmduncan@uark.edu (H.D.); jhesteki@uark.edu (J.H.); 2 Division of Nephrology, Department of Medicine, David Geffen School of Medicine, University of California, Los Angeles, CA 90095, USA; ikurtz@mednet.ucla.edu

**Keywords:** nanofiltration, flux, glucose, urea, kidney disease, monovalent ion, divalent ions

## Abstract

End-stage renal disease occurs when there is permanent loss of the kidney’s ability to filter toxins from the blood. Due to the limited number of transplants, dialysis is currently the most common treatment, but it significantly limits a patient’s lifestyle and has significant side effects. One solution is an artificial kidney, but significant challenges remain in its development. One challenge is the separation of glucose from urea. Nanofiltration is ideal for this separation; however, there is little understanding of the important parameters for this separation under physiological conditions. In this study, operating parameters (pressure and temperature) as well as feed conditions (increased glucose/salt) were explored for their effects on the separation of glucose from urea in six commercial membranes. The rejection of monovalent and divalent ions was also characterized. While increasing pressure increased flux, it had little effect on metabolite rejection, except for glucose, which increased above 20 psi. Increasing temperature led to a slight increase in flux and a slight decrease in the rejection of divalent ions. Glucose rejection was sensitive to feed conditions, while urea rejection was less affected. Divalent ions were rejected more strongly than monovalent ions and were also more affected by feed conditions.

## 1. Introduction

Greater than 35 million Americans have kidney disease [1]. End-stage renal disease (ESRD) is characterized by a permanent loss in the kidney’s ability to filter out toxins in the blood [2]. Currently, the only treatment options for patients with ESRD are either kidney transplantation or dialysis. Patients may wait for up to 10 years for a donor kidney, therefore leaving no other option for treatment but dialysis. Unfortunately for patients, undergoing dialysis causes physical pain, social issues due to treatment times up to 15 h per week, and significant financial burdens [3]. Medicare beneficiaries paid over USD 37 billion for treating ESRD in 2020. With all these struggles, including a lowered lifespan of between five to ten years after diagnosis, replacements for dialysis are crucial [4,5]. Although dialysis has been the primary treatment for ESRD aside from kidney transplants, there exist several limitations. The two methods of dialysis (hemodialysis or peritoneal) both exhibit the same limitation of utilizing passive separation as a means of separating limited metabolites and toxins, leaving several metabolites untouched by the dialysis process [6]. All in all, even though dialysis prolongs survival while patients await a kidney that may never come, future solutions are needed to address these limitations.

Blood chemistry is extraordinarily complex and requires an equally complex system to separate a wide range of compounds that differ in several ways, including molecular size and charge. Glucose has a molecular weight of 180.2 g/mol, while urea is 60.1 g/mol. The relative cations explored were Na^+^, K^+^, Mg^2+^, and Ca^2+^, all of which have even lower molecular weights (22.99, 39.10, 24.31, and 40.08 g/mol, respectively) [7]. While ultrafiltration membranes are often used in applications such as dialysis, nanofiltration (NF) is more suitable for species within this size range [8,9,10]. NF has the capacity to address the medium-sized metabolites, while dialysis has no active transport but uses dialysate to induce diffusion across the dialyzer [11,12]. In recent work by Hestekin et al., they proposed an artificial kidney in which nanofiltration was a key component for the separation of glucose from urea [13]. Among the separation requirements for an improved treatment of kidney disease are high glucose rejection and low urea rejection. NF also possesses the ability to allow high passage of monovalent ions while highly rejecting divalent ions. The properties of the membranes, such as pore size, membrane active materials, and/or composition along with the properties of the solution, affect the separation of both monovalent and divalent ions [14,15,16,17,18]. To obtain an understanding of an NF membrane’s ability to reject salts, companies provide what is called a nominal salt rejection. However, the reported rejection is only for the reference ion (MgSO_4_), and each company’s protocol can vary with regard to the concentration of MgSO_4_, pressure, running time, and flowrates [19].

NF has been applied to a wide range of applications, including many with similar solute size to those of physiological interest [20,21,22,23,24]. However, there is a need to better understand the performance of NF membranes under physiological conditions if they are to be used for applications such as the treatment of ESRD. Specifically, the ability of NF membranes to separate metabolites contained within plasma, such as glucose, urea, and cations (Na^+^, K^+^, Mg^2+^, and Ca^2+^), needs to be evaluated under varying operating parameters and physiological feed variations. Membrane performance parameters such as flux and metabolite rejection were used to evaluate six commercial membranes (NF90, NF270, NF3, NF4, dnf40, and dnf80) for their ability to separate metabolites usually filtered by the kidney.

Many ESRD patients will experience fluctuations in their blood concentrations of sugar and salt. Hyperglycemia occurs when a patient’s blood glucose exceeds 125 mg/dL and is a life-threatening condition that can occur in patients with diabetes [25]. Hyperkalemia occurs when a patient’s blood potassium exceeds 5.2 mmol/L (~20 mg/dL) and has been reported to occur at a higher frequency in patients with diabetes than healthy individuals [26]. Therefore, this study also investigated how increased glucose and potassium ion concentrations in the blood might influence NF performance.

## 2. Materials and Methods

### 2.1. Commercial NF Membranes and Their Characteristics

Six different commercial membranes were tested. NF90 and NF270 from Dupont FilmTec (Edina, MN, USA), NF4 and NF3 from Axeon Water Technologies (Temecula, CA, USA), and dnf40 and dnf80 from NX Filtration (Enschede, The Netherlands) were selected for testing. Dnf40 and dnf80 are hollow fiber, thin-film composite (TFC) semi-permeable membranes, while the other four are spiral wound TFC. NF Dupont membranes (NF90 and NF270) were selected due to their prevalence in the literature, while Axeon membranes (NF3 and NF4) and NX Filtration (dnf40 and dnf80) membranes were less common in the literature. NF90 and NF270 were selected to determine if membrane composition played a role in separation. NF3 and NF4 varied only in their nominal salt rejections, while dnf40 and dnf80 had different nominal salt rejections. Table 1 lists the membranes and their relevant characteristics, including membrane composition, active area, and nominal salt rejection. Nominal salt rejections were provided by the companies and reflect the rejection of MgSO_4_ under each company’s standard set of conditions. For Dupont membranes (NF90 and NF270), these tests were based on 2000 ppm MgSO_4_ at 25 °C and 70 psi. For Axon (NF4 and NF3), these tests were performed at 550 ppm MgSO_4_ at 25 °C and 70 psi. For NX Filtration (dnf40 and dnf80), these tests were performed at 0.6 ppm MgSO_4_ at 25 °C and ~44 psi.

### 2.2. Feed Conditions of Commercial NF Membrane Experiments

Three different feed conditions (healthy, hyperglycemic, and hyperkalemic) were tested for each membrane based on the physiological blood concentration of key metabolites (glucose, urea, Na^+^, K^+^, Mg^2+^, and Ca^2+^). Glucose and urea were purchased from Sigma Aldrich (St. Louis, MO, USA). Sodium chloride, potassium chloride, magnesium sulfate heptahydrate, and calcium chloride were obtained from VWR (Radnor, PA, USA), Amresco (Solon, OH, USA), and Sigma Alrich (St. Louis, MO, USA), respectively. The healthy feed solution was based on the blood concentrations in a healthy person and was as follows: 100 mg/dL glucose, 10 mg/dL urea, 321.8 mg/dL sodium, 2.0 mg/dL magnesium, 15.6 mg/dL potassium, and 9.5 mg/dL calcium. The hyperglycemic feed had the same composition as the healthy feed, except that the glucose concentration was doubled to 200 mg/dL. The hyperkalemic feed had the same composition as the healthy feed, except that the potassium concentration was increased to 31.3 mg/dL.

### 2.3. Bench-Scale Unmodified Commercial Membrane Experimental Set-Up and Procedure

Six commercial NF membranes were run via continuous circulation of both the permeate and retentate recycled into the feed. The volumetric feed flowrate was constant for all experiments at 100 mL/min. Pressure and flow were maintained across the membranes by using a valve and microgear pump. Pressure was varied at 20, 40, and 60 psi at ~22 °C for each membrane. Nitrogen gas from Airgas (Radnor, PA, USA) was acquired to maintain the pressure of the system. Feed conditions (healthy, hyperglycemic, and hyperkalemic) were tested for each of the six membranes. A stirring rod with stirring plate (Sterlitech, Auburn, WA, USA) was used to maintain constant mixing. As outlined in Table 1, the NF membranes were of different sizes, making it necessary to use different volumes to flush the membrane with feed solution. For the larger membranes, NF90 and NF270, 2000 mL of feed solution was allowed to pass through the membrane and then discarded. These membranes maintained a volume of about 800 mL. For NF3 and NF4, 1000 mL of feed solution was passed through the membrane and then discarded. These membranes maintained a volume of 800 mL as well. For the smallest membranes, dnf40 and dnf80, 1000 mL of feed solution was passed through the membrane and then discarded. These membranes maintained a volume of about 500 mL. Depending on the application, NF membranes are often run at hundreds to thousands of psi, so the pressure was set to 20, 40, and 60 psi to explore physiological conditions. It took 30 min at each pressure for the system to reach equilibrium. At the end of each 30 min, samples were collected from the feed, permeate, and retentate streams. Flowrates of the permeate and retentate streams were also recorded. For experiments examining the effect of temperature, the membranes with the smallest area (dnf40 and dnf80) were tested due to their small size. For these experiments, the temperature of the feed solutions was controlled at 37 °C using a Southwest Science 20 L general-purpose water bath (San Antonio, TX, USA), with insulation (Milwaukee, WI, USA) to keep heat from being lost through the tubing and sides of the bottle, and a VWR hot plate with magnetic stirrer to keep the feed solution thoroughly mixed and hot.

### 2.4. Chemical Analysis

Samples from all experimental sets were analyzed for solute concentrations in the same way. The concentrations of glucose were determined using Glucose (HK) Assay Kits obtained from Sigma Alrich (St. Louis, MO, USA). For the glucose assay, a sample of 10 µL was mixed with 190 µL glucose assay reagent. After being mixed for 15 min, a UV plate reader was utilized at 340 nm absorbance to determine the glucose concentration. Data were collected for the feed, permeate, and retentate. The concentrations of urea were determined using an assay kit from BioAssay Systems (Hayward, CA, USA) called QuantiChrom Urea Assay Kit II. For the urea assay kit, 20 µL of sample was mixed with 85 µL urea assay reagent. After being mixed for 5 min, a UV plate reader was utilized at 557 nm absorbance to determine urea concentration. To calculate the concentrations based on the optical density, calibration curves for each assay kit were performed at 0 mg/dL, 30 mg/dL, 60 mg/dL, and 90 mg/dL. Samples were analyzed on a Thermo Scientific ICS-6000 ion chromatography system (Bremen, Germany) equipped with an AS-AP autosampler and automatic eluent generator. Ions were separated using a CG16 column (4 mm-250) with 30 µM methyl sulfonic acid. Sample quantitation was performed using external calibration of a six cation mixed standards (High-Purity Standards IC-CAT6-1-100) (North Charleston, SC, USA). The four standard cations of interest were 20 mg/dL sodium, 50 mg/dL potassium, 25 mg/dL magnesium, and 50 mg/dL calcium. These were diluted 10×, 100×, and 1000× to create a standard curve for ion concentrations. Ion samples were diluted 1:10 with distilled water before they were tested.

### 2.5. Rejection

To gain an idea of the performance of each membrane, rejection was measured at varying pressures of 20, 40, and 60 psi for the commercial TFC NF membranes. The rejection (R) of each solute was calculated using Equation (1).

(1) $$R_{i}=\frac{\left(C_{i,f}-C_{i,p}\right)}{C_{i,f}}\;*\;100\%$$

where C_i,f_ is the feed concentration of species i and C_i,p_ is the permeate concentration of species i.

### 2.6. Statistical Analysis

Statistical analysis was performed on the permeate fluxes and glucose, urea, and cation (Na^+^, K^+^, Mg^2+^, and Ca^2+^) rejections via standard deviation to determine significance of findings. Pressure, temperature, and feed concentration condition changes as well as comparing the membranes against each other were among the variables tested. All experiments were conducted in triplicate (N = 3). Unpaired *t*-tests using the GraphPad QuickCalcs (Graphpad Software Inc., San Diego, CA, USA) were also conducted for all experiments in order to verify their statistical significance. Results were reported as significantly or statistically different for *p* < 0.05.

## 3. Results and Discussion

### 3.1. Permeate Flux of NF Commercial Membranes Based on Feed Conditions

Figure S1 shows the effect of pressure on permeate flux (Jp) for all commercial NF membranes tested under the three different feed conditions. As expected, the flux generally increased with increasing pressure. NF4 had the lowest flux, with an average value of 0.007 L/m^2^ × h at 20 psi and 0.016 L/m^2^ × h at 60 psi. NF90 also had a low flux, with an average value of 0.015 L/m^2^ × h at 20 psi and 0.065 L/m^2^ × h at 60 psi. NF3 had the highest average flux of 0.13 L/m^2^ × h at 20 psi and 0.42 L/m^2^ × h at 60 psi. NF270 also had a high flux, with an average value of 0.13 L/m^2^ × h at 20 psi and 0.35 L/m^2^ × h at 60 psi. Both dnf40 and dnf80 had a flux around 0.1 L/m^2^ × h at 20 psi, which rose to ≥0.3 L/m^2^ × h at 60 psi.

While it is known that increasing pressure generally increases flux, the effect of small, physiological changes in the feed concentrations on flux was unknown. Depending on the health of a patient, their blood sugar and salt concentrations can change. To understand these effects, this study included hyperglycemic and hyperkalemia feed conditions. Hyperglycemia occurs in patients who have high blood sugar, even up to double the normal value. This is often seen in patients with diabetes. Hyperkalemia occurs in patients who have high levels of potassium in their blood, which can exceed double the normal value. These conditions can also be seen in patients with diabetes. Figure S2 shows the flux values for each membrane under the different feed conditions at the three different pressures. In general, the feed composition had no effect on permeate flux, which seems reasonable given that the feed conditions are still relatively similar. However, feed conditions did influence flux for the two small polysulfone membranes, dnf40 (Figure S2E) and dnf80 (Figure S2F). For dnf40, the fluxes differed significantly for all feed conditions, with healthy having the highest flux and hyperkalemic having the lowest flux. Similarly, dnf80 also differed for all feed conditions, with healthy generally having the highest flux and hyperglycemic usually having the lowest flux. Although our study did not find any correlation between permeate flux and feed concentration changes, in studies with greater changes in the feed composition, a dramatic decrease in permeate flux as the feed concentration increased has been reported [28,29].

Temperature is known to increase flux through reducing solution viscosity and opening membrane pores [30,31]. Figure S3 compares the permeate flux at room temperature (~22 °C) and physiological temperature (37 °C) for dnf40 and dnf80 in hyperglycemic feed conditions at the three different pressures. These membranes were selected due to their small size, allowing a more consistent temperature to be maintained. As shown in Figure S3, for dnf40, there was no statistical difference between the fluxes due to the temperature at any pressure. For dnf80, there was an increase of 35–50% in the flux at physiological temperature compared to room temperature. This difference was statistically significant (*p* ≤ 0.005) for all pressures. This indicates that temperature effects can be membrane-specific, highlighting the importance of testing under the exact intended operating conditions for the maximum accuracy.

### 3.2. Urea Rejection of Commercial NF Membranes at Varying Feed Conditions

**Figure 1:**
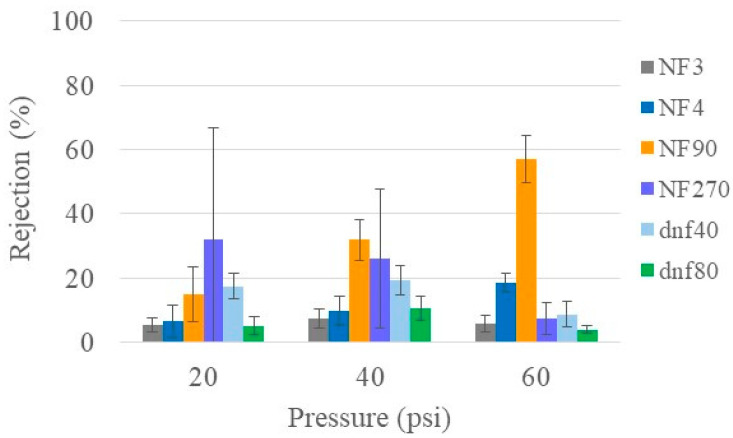
Urea rejection of all commercial membranes at varying pressures for healthy feed at 22 °C.

Urea is one of the primary toxins that the kidneys are responsible for removing. Therefore, the desirable membrane will have the ability to remove a large amount of urea, which, for the purpose of this study, corresponds to low rejection. Figure 1 shows the urea rejection of all six NF membranes at varying pressures for healthy feed conditions. NF3 and dnf80 had the lowest average rejection of urea (≤10%) at all pressures. Although NF270 had average rejection values up to 35%, the variability was so large that it was hard to draw a definitive conclusion. NF4 and dnf40 had average rejections that ranged from ~5 to 20% depending on the pressure. NF90 showed the most significant change in rejection with pressure as well as the largest average rejection (57%). Similarly, low urea rejection values have also been reported in the literature. Shao et al. obtained a urea rejection of ~20% [8]. Ray et al. had urea rejection values below 50% and found that the lowest values occurred at higher pH (12.5 vs. 5) [32]. Courtney and Randall observed a urea rejection of <60% for NF90 and < 15% for NF270 in both simulated and real urine, which are similar to the values observed in this study [33].

**Figure 2:**
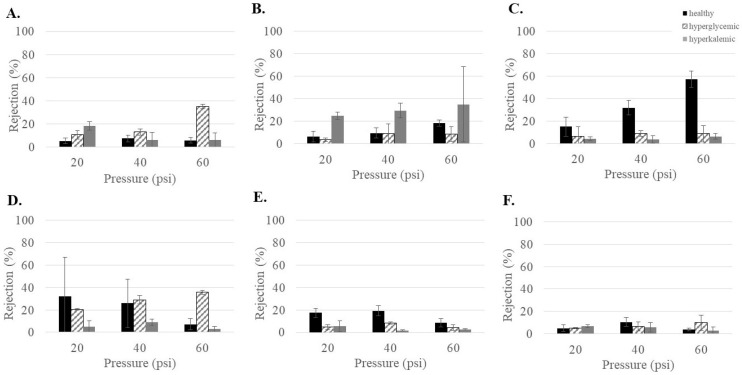
Urea rejection as a function of feed conditions for the commercial membranes (**A**) NF3, (**B**) NF4, (**C**) NF90, (**D**) NF270, (**E**) dnf40, and (**F**) dnf80.

The health of the patient can lead to variations in blood concentration. In patients with diabetes, this can include hyperglycemic (high blood sugar) and hyperkalemic (high blood potassium). Therefore, it was important to understand what influence these increased concentrations might have on urea rejection. As can be seen in Figure 2, the effects were highly variable and membrane-dependent. NF3 and NF270 showed increased urea rejection for hyperglycemic conditions at 60 psi. NF4 showed increased urea rejection for hyperkalemic conditions at low pressures (20 and 40 psi). NF90 had decreased rejection of urea under both hyperglycemic and hyperkalemic conditions at 40 and 60 psi. Urea rejection decreased under both hyperglycemic and hyperkalemic conditions at 20 and 40 psi for dnf40. No effects due to feed conditions were observed for dnf80. Overall, the changes in urea rejection appeared to be very membrane specific. Since low urea rejection was desirable, dnf40 and dnf80 had the overall best performance. However, NF90 was notable in that it showed low urea rejection for the hyperglycemic and hyperkalemic feeds, which are conditions that can occur in patients undergoing dialysis.

Since temperature is known to influence solution viscosity and membrane pore structure, its effect on urea rejection was evaluated. Figure S4 compares the glucose rejection at room temperature (~22 °C) and physiological temperature (37 °C) for dnf40 and dnf80 in hyperglycemic feed conditions at the three different pressures. For all conditions, there did not appear to be any change in urea rejection due to temperature.

### 3.3. Glucose Rejection of Commercial NF Membranes at Varying Feed Conditions

**Figure 3:**
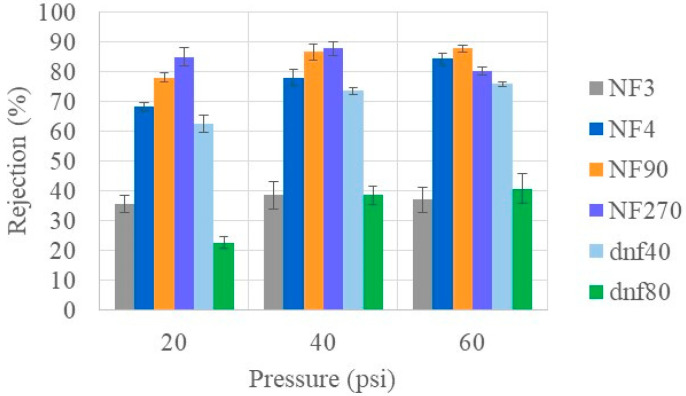
Glucose rejection of all commercial membranes at varying pressures for healthy feed at 22 °C.

In addition to the removal of urea, it is important to return glucose to the patient. Glucose is an important source of energy for the body, and too much sugar in urine can lead to infections. Figure 3 shows the glucose rejection at varying pressures using the healthy feed condition for the commercial membranes tested. Most of the membranes had a high glucose rejection, especially at high pressure. However, NF3 and dnf80 showed maximum average rejections ≤40% at all pressures, indicating they would not be effective membranes for glucose rejection. While many of the membranes showed a statistical increase in rejection when going from 20 to 40 psi, most of the membranes had the same rejection statistically at 40 and 60 psi. The notable exceptions were NF4, which showed a statistically significant increase with each increase in pressure, and NF270, which showed a statistical decrease from 40 to 60 psi. Interestingly, the rejection differences between the commercial membranes decreased as the pressure increased. NF90 (at 40 and 60 psi) and NF270 (at 40 psi) had the highest overall rejection values, approaching 90%. Mohammad et al. found that at higher pressures (>40 psi), the glucose retention became similar [34].

**Figure 4:**
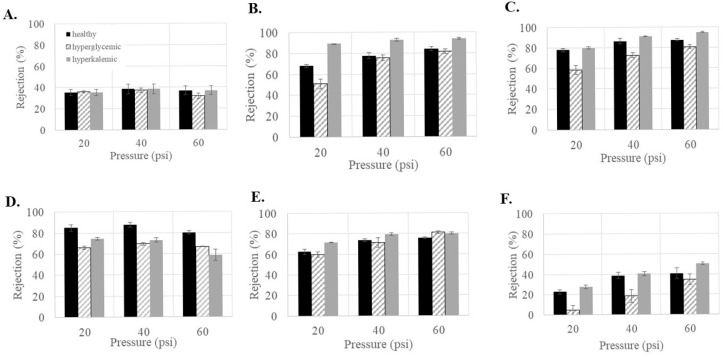
Glucose rejection as a function of feed conditions for the commercial membranes (**A**) NF3, (**B**) NF4, (**C**) NF90, (**D**) NF270, (**E**) dnf40, and (**F**) dnf80.

Since the patient’s health can affect factors such as the concentration of sugar (hyperglycemic) and ions (hyperkalemic) in the blood, it was important to see if the glucose rejections would be altered under those conditions. As shown in Figure 4, feed conditions influenced the glucose rejection for all membranes except NF3 (Figure 4A). In general, increasing the concentration of glucose (hyperglycemic feed) decreased the ability of the membranes to reject glucose. This was especially noticeable in NF270 (Figure 4D), where the average rejection decreased by 10–20% compared to under healthy feed conditions. NF4 and NF90 also showed decreased rejection but were able to maintain a glucose rejection >80% at 60 psi. In general, higher glucose concentration in the feed appeared to decrease the rejection of glucose in the commercial NF membranes, which is consistent with other observations in the literature [35,36]. Vellenga and Tragardh found that the NF retention of sucrose was not affected by an increase in concentration but that the increased concentration did lead to a significant increase in the concentration polarization [37].

Alternatively, increasing the concentration of salt (hyperkalemic feed) generally increases the rejection of glucose. This was especially noticeable in NF4 (Figure 4B), where the average rejection increased by 10–20% compared to under healthy feed conditions. NF90 and dnf80 also showed increased rejection, with NF90 having a rejection around 95% at 60 psi. Interestingly, NF270 was the only membrane that showed a decrease in rejection of 10–20%. Several studies have found that glucose retention decreased with increasing NaCl concentration [38,39]. Nilsson et al. found that increasing the KCl concentration decreased glucose retention [40]. These results differ from this study and may be due to the fact that much larger variations in salt concentrations were used in these studies than in this study.

In terms of glucose rejection, the membranes were influenced by pressure and feed conditions in their own ways. In terms of glucose rejection, NF3 and dnf80 both had very low rejections that would not be suitable for returning glucose to a patient’s body. While NF270 had high rejections under healthy feed conditions, its performance decreased under both the hyperglycemic and hyperkalemic feed conditions, making it unsuitable. Of all the membranes tested, NF90 had the most consistently high glucose rejection, especially at 60 psi, and, therefore, would be the best-suited membrane for returning glucose to the patient.

**Figure 5:**
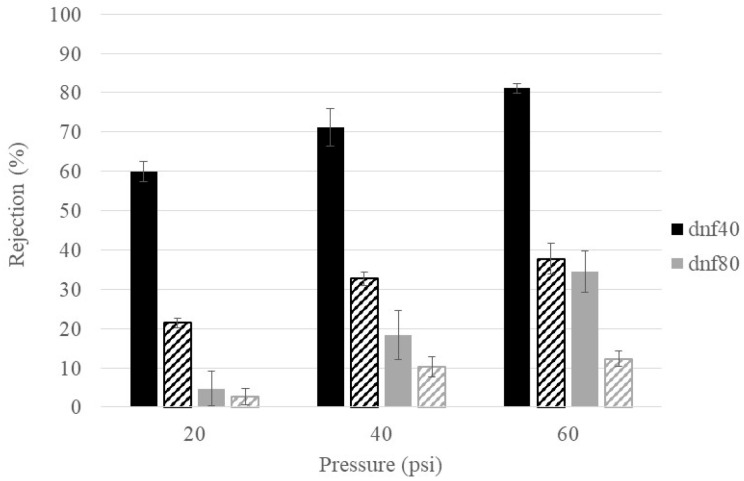
Temperature effects on glucose rejection for dnf40 and dnf80. Color indicates the membrane (black = dnf40, gray = dnf80) and texture indicates the temperature (solid = room, striped = physiological).

Temperature is known to influence solution viscosity and membrane pore structure, so it was important to determine its influence on glucose rejection. Figure 5 compares the glucose rejection at room temperature (~22 °C) and physiological temperature (37 °C) for dnf40 and dnf80 in hyperglycemic feed conditions at the three different pressures. For dnf80, there was no statistical difference between the rejection at the two temperatures, except at 60 psi, where the rejection decreased from an average value of ~35% to ~12%. For dnf40, the increased temperature caused the glucose rejection to be reduced by at least 50% at all pressures. A decrease in the rejection of metabolites when temperature increases has also been observed in the literature. Freger et al. saw this behavior for the rejection of lactate, which is derived from glucose, in nanofiltration membranes [41]. Bandini and Morelli found that increased temperatures decreased the rejection of monosaccharides and disaccharides via nanofiltration membranes [42]. While increased temperature often decreases sugar rejection, its effect can be membrane- and condition-specific and should be considered when evaluating membranes for physiological applications.

### 3.4. Ion Rejections of Commercial NF Membranes at Varying Feed Conditions

**Figure 6:**
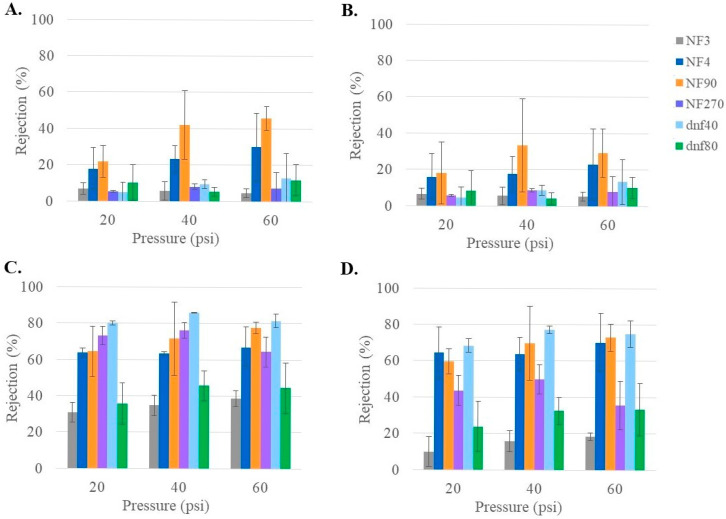
Rejection of ion (**A**) Na^+^, (**B**) K^+^, (**C**) Mg^2+^, and (**D**) Ca^2+^ for all commercial membranes at varying pressures for healthy feed at 22 °C.

While ion rejection is not the primary goal of this study, NF membranes have previously been reported to have a higher rejection of divalent than monovalent ions [43,44]. Since maintaining an appropriate ion concentration is important for patient health, it was valuable to understand how these membranes might influence the ion concentration. The ion rejection of each of the six membranes (NF90, NF270, NF4, NF3, dnf40, and dnf80) using the healthy feed condition is summarized in Figure 6. The rejections for the monovalent ions (Na^+^ in 6A and K^+^ in 6B) averaged below 30% for every membrane, except NF90. The rejections for the divalent ions (Mg^2+^ in 6C and Ca^2+^ in 6D) were >40% for all membranes except NF3 and dnf80. For NF90 and NF270, the divalent rejections were often >70%. Pressure did not seem to have much impact on ion rejection, although this was difficult to determine due to the variability within the data. The low ion rejections of NF3 and dnf80 and the high ion rejections of NF90 and NF270 agreed with the pattern observed from their nominal rejections in Table 1. A study by Pages et al. examined the rejection of dominant and trace monovalent and divalent ions for NF270 [45]. When observing the dominant salts, they found that the rejection of magnesium was typically ~20% higher than for sodium, which is consistent with the current study.

The effect of feed conditions (hyperglycemic, hyperkalemic) on ion rejection was also examined. In general, the monovalent ions (Figures S5 and S6) showed very little or no differences in rejection with the varying feed conditions. For divalent ions (Figures S7 and S8), the feed conditions did not have a significant effect on rejection for NF3, NF270, dnf40 or dnf80. For NF4 and NF90, the hyperglycemic and hyperkalemic feed conditions increased divalent ion rejection, as indicated in Figures S7 and S8. The hyperglycemic and hyperkalemic feed conditions had the most notable effect on increasing the rejection of Mg^2+^ for all pressures in the NF4 membrane compared to the healthy feed condition.

The effect of temperature on ion rejection was also evaluated using the dnf40 and dnf80 membranes. The monovalent ion (Na^+^, K^+^) rejections did not show any significant changes between room and body temperature (22 °C vs. 37 °C), as shown in Figure S9A,B. For the divalent ions, dnf80 did not show any significant difference in ion rejection with temperature, as can be seen in Figure S9C,D. For the dnf40 membrane, increasing the temperature did significantly decrease rejection in both divalent ions (Figure S9C,D) at 20 and 60 psi. This effect might also have been observed at 40 psi, but the variability was too large to be considered significant. Overall, the rejection of monovalent ions was not affected by the temperature change nor the rejection of divalent ions by dnf80. However, for dnf40, temperature did seem to have a significant effect on Mg^2+^ and Ca^2+^ rejection in some cases. The literature has also reported a decrease in ion rejection with increasing temperature, although the temperature differences examined are often much larger than those in this study. Nilsson et al. found that the rejection of KCl decreased with increasing temperature (from 20 °C to 50 °C) for the NF membrane NFT-50 [40]. They attributed part of this change to swelling in the membrane. Ben Amar et al. also observed decreased rejection of NaCl, which increased from 22 °C to 50 °C, and found that it correlated with an increased effective pore radius in the membrane [46].

## 4. Conclusions

This study explored the use of NF membranes for the separation of small metabolites (urea and glucose) as well as monovalent versus divalent ions for the treatment of ESRD. The effect of operating parameters such as temperature and pressure was explored as well as the effects of feed compositions due to patient health. While increasing the pressure increased the flux, it generally did not impact the transport of metabolites. The only exception was that an increase in glucose rejection was observed in increasing the pressure from 20 to 40 psi but with no further improvement when increased to 60 psi. Increasing the temperature led to a slight increase in flux as well as a slight decrease in the rejection of divalent ions. Other effects were either negligible or too membrane-specific to allow for a general conclusion. The effect of increasing glucose or potassium ion concentrations on urea rejection was often minimal, with the exceptions being membrane specific. Glucose rejection decreased with increased glucose concentration and increased with increased potassium ion concentrations. The rejection of monovalent ions was generally low and negligibly influenced by either operating parameters or feed conditions. Divalent ion rejection did show some changes based on feed concentrations, with a general increase in rejection in the unhealthy feed conditions and decreased rejection with increasing temperature.

For the treatment of ESRD, a membrane with low urea rejection and high glucose rejection was desired. NF3 and dnf80 had the lowest average rejection of urea (≤10% at all pressures); however, they also had the lowest average rejections of glucose (≤40% at all pressures). NF4 and dnf40 had average urea rejections that ranged from ~5 to 20% depending on the pressure and reasonably high average glucose rejections of >75% at 60 psi for all feed conditions. NF270 had average urea rejection values up to 35%, with high variability. In addition, NF270 had low glucose rejections under hyperglycemic feed conditions. While NF90 had a high urea rejection, this value also decreased the most significantly (<10% at 60 psi) when under the unhealthy (hyperglycemic and hyperkalemic) feed conditions. NF90 had very high glucose rejection values, approaching 90% (at 40 and 60 psi). Based on overall performance, NF4 and dnf40 had the best combination of low urea rejection and high glucose rejection. If the performance under unhealthy feed conditions is considered, then NF90 also shows promise as a membrane for application in the treatment of ESRD. Overall, as can be seen from this study, the operating parameters (pressure and temperature) as well as the feed conditions (high sugar or high salt) can have an influence on membrane performance and should be considered for any application.

## Figures and Tables

**Table 1:** Commercially available nanofiltration membranes and their characteristics.

Membrane Name	Membrane Composition	MWCO (Da)	Active Area (m^2^)	Nominal Salt Rejections	Manufacturer
NF3	polyamide	ND	1.3	~45%	Axeon
NF4	polyamide	ND	1.3	~85%	Axeon
NF90	polyamide	180 Da	2.6	97.0%	Dupont
NF270	polypiperazine	340 Da	2.6	>97.0%	Dupont
dnf40	polysulfone	400 Da	0.05	91%	NX Filtration
dnf80	polysulfone	800 Da	0.05	76%	NX Filtration

ND = not determined or found in literature. Molecular weight cutoff (MWCO) for NF90 and NF 270 from Lopez-Munoz et al. [27]; MWCO for dnf40 and dnf80 and nominal salt rejections for all membranes from manufacturer specifications.

## Data Availability

The datasets used and/or analyzed during the current study may be made available by the corresponding author upon reasonable request.

## Supplementary Materials

The following supporting information can be downloaded at: https://www.mdpi.com/article/10.3390/membranes15060168/s1, Figure S1. Permeate flux of all commercial membranes at various pressures for healthy feed at 22 °C; Figure S2. Permeate flux as a function of feed conditions for the commercial membranes (A) NF3, (B) NF4, (C) NF90, (D) NF270, (E) dnf40, and (F) dnf80; Figure S3. Temperature effects on permeate flux for dnf40 and dnf80; Figure S4. Temperature effects on urea rejection for dnf40 and dnf80; Figure S5. Sodium ion (Na^+^) rejection as a function of feed conditions for the commercial membranes (A) NF3, (B) NF4, (C) NF90, (D) NF270, (E) dnf40, and (F) dnf80; Figure S6. Potassium ion (K^+^) rejection as a function of feed conditions for the commercial membranes (A) NF3, (B) NF4, (C) NF90, (D) NF270, (E) dnf40, and (F) dnf80; Figure S7. Magnesium (Mg^2+^) rejection as a function of feed conditions for the commercial membranes (A) NF3, (B) NF4, (C) NF90, (D) NF270, (E) dnf40, and (F) dnf80; Figure S8. Calcium (Ca^2+^) rejection as a function of feed conditions for the commercial membranes (A) NF3, (B) NF4, (C) NF90, (D) NF270, (E) dnf40, and (F) dnf80; Figure S9. Temperature effects on ion rejection for dnf40 and dnf80 for (A) Na^+^, (B) K^+^, (C) Mg^2+^, and (D) Ca^2+^.

## Abbreviations

The following abbreviations are used in this manuscript:

ESRD	End-stage renal disease
MWCO	Molecular weight cutoff
NF	Nanofiltration
TFC	thin-film composite
