# Supplementary material for: Exploring Nanofiltration for Transport of Small Molecular Species for Application in Artificial Kidney Devices to Treat End-Stage Kidney Disease
Source: Membranes (Basel). 2025 Jun 2;15(6):168. doi: 10.3390/membranes15060168 (PMC12195112; doi:10.3390/membranes15060168)
Supplement: Supplementary file 1 [file membranes-15-00168-s001.zip › membranes-3648077-supplementary.pdf]

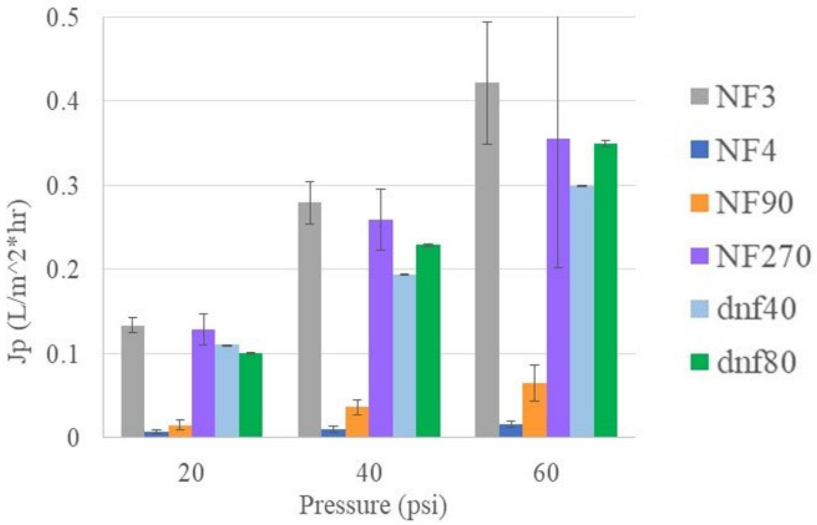

Figure S1. Permeate flux of all commercial membranes at various pressures for healthy feed at 22°C.

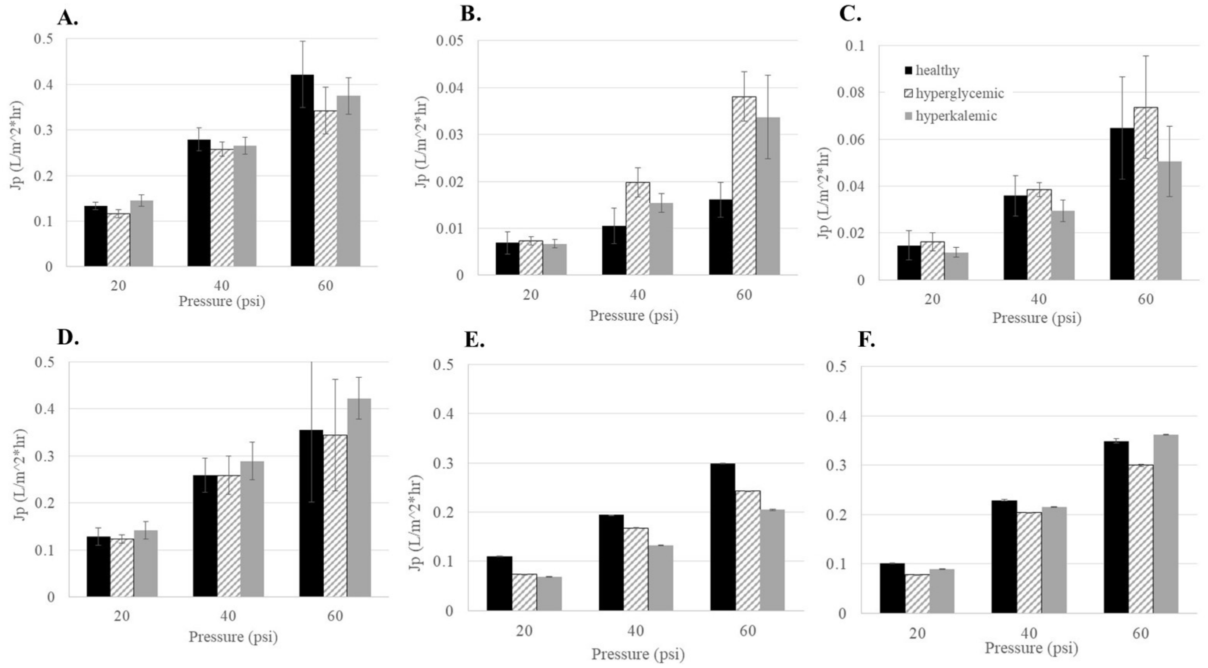

Figure S2. Permeate flux as a function of feed conditions for the commercial membranes (A) NF3, (B) NF4, (C) NF90, (D) NF270, (E) dnf40, and (F) dnf80.

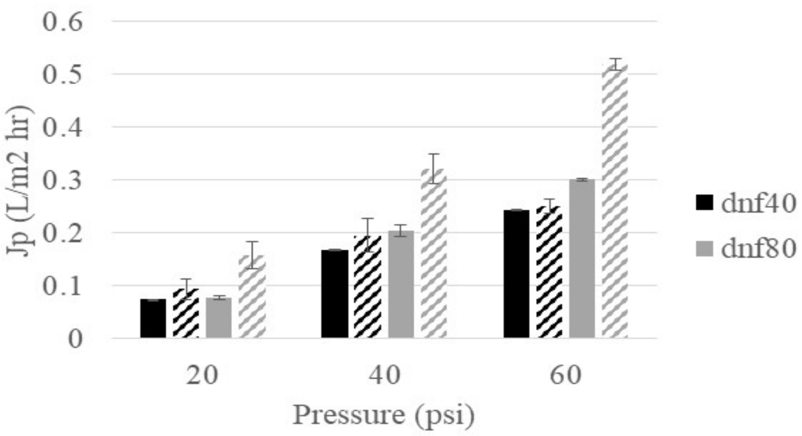

+

Figure S3. Temperature effects on permeate flux for dnf40 and dnf80. Color indicates the membrane (black = dnf40, gray = dnf80) and texture indicates the temperature (solid = room, striped = physiological).

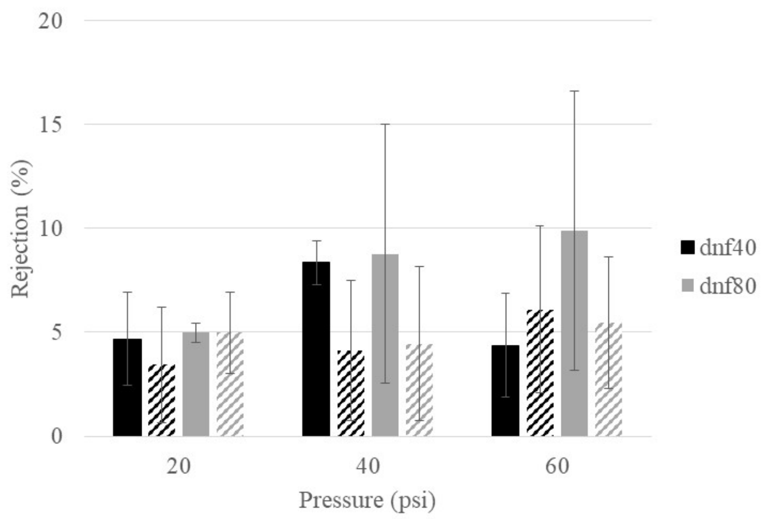

Figure S4. Temperature effects on urea rejection for dnf40 and dnf80. Color indicates the membrane (black = dnf40, gray = dnf80) and texture indicates the temperature (solid = room, striped = physiological).

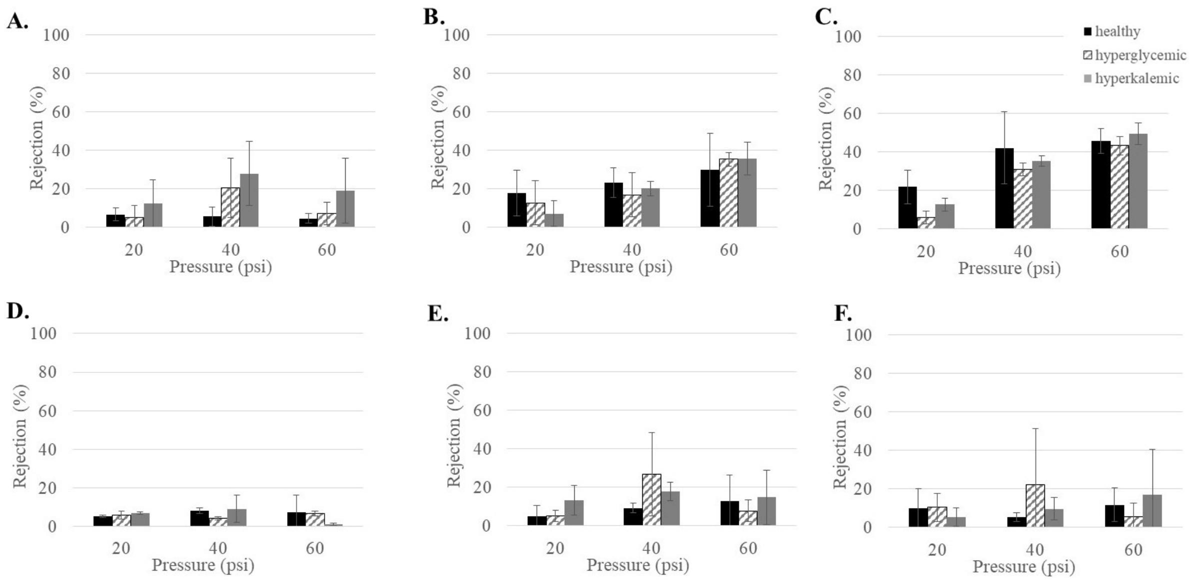

Figure S5. Sodium ion (Na^+) rejection as a function of feed conditions for the commercial membranes (A) NF3, (B) NF4, (C) NF90, (D) NF270, (E) dnf40, and (F) dnf80.

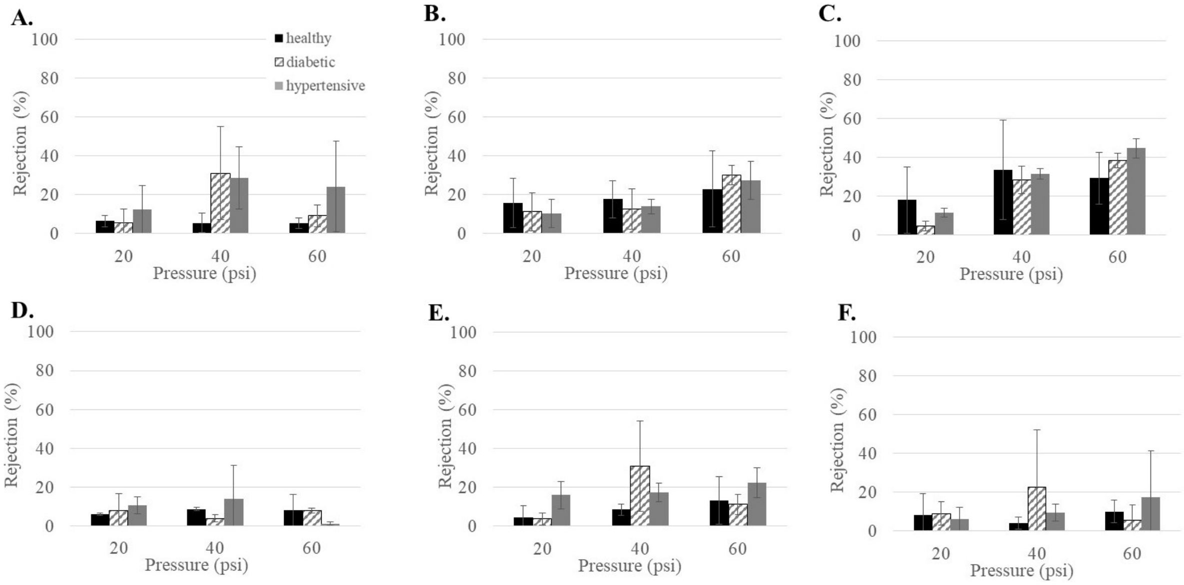

Figure S6. Potassium ion (K^+) rejection as a function of feed conditions for the commercial membranes (A) NF3, (B) NF4, (C) NF90, (D) NF270, (E) dnf40, and (F) dnf80.

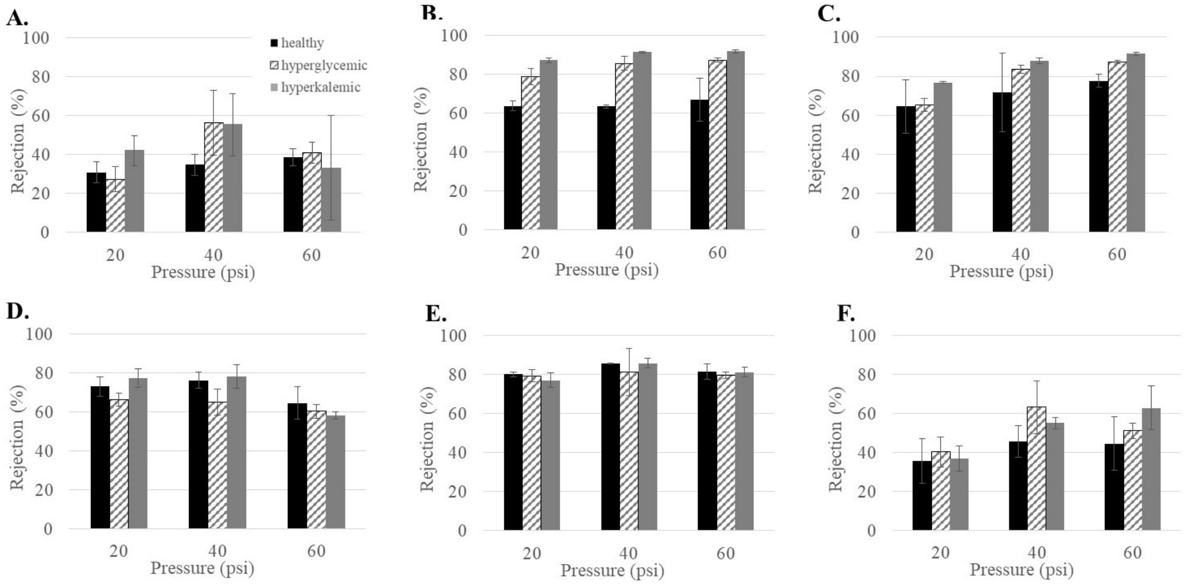

Figure S7. Magnesium (Mg^{2+}) rejection as a function of feed conditions for the commercial membranes (A) NF3, (B) NF4, (C) NF90, (D) NF270, (E) dnf40, and (F) dnf80.

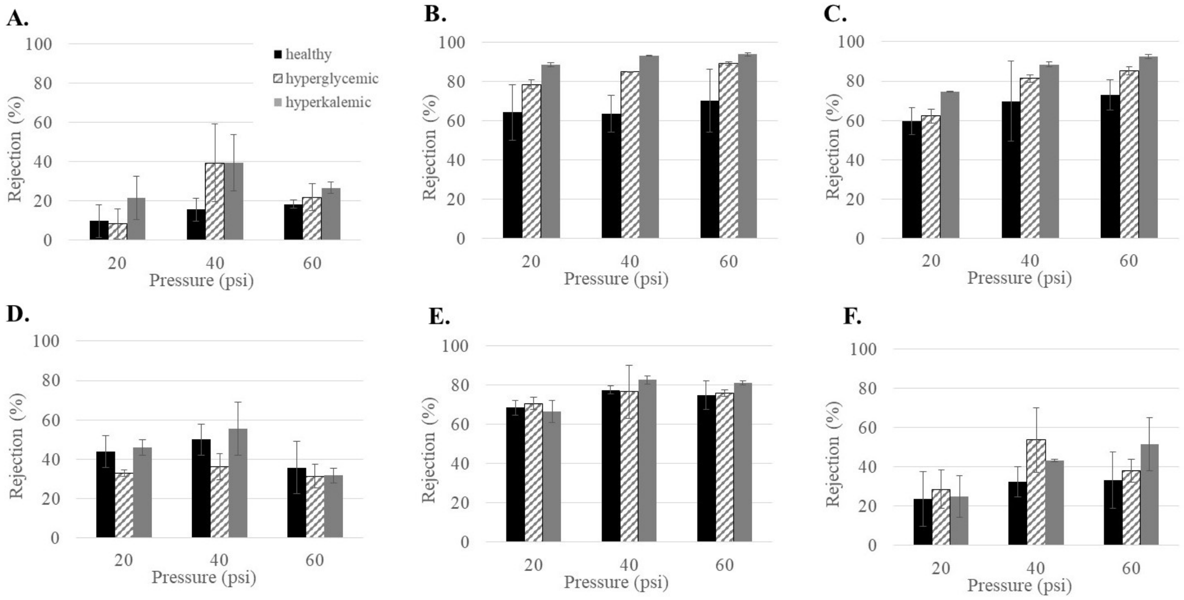

Figure S8. Calcium (Ca^{2+}) rejection as a function of feed conditions for the commercial membranes (A) NF3, (B) NF4, (C) NF90, (D) NF270, (E) dnf40, and (F) dnf80.

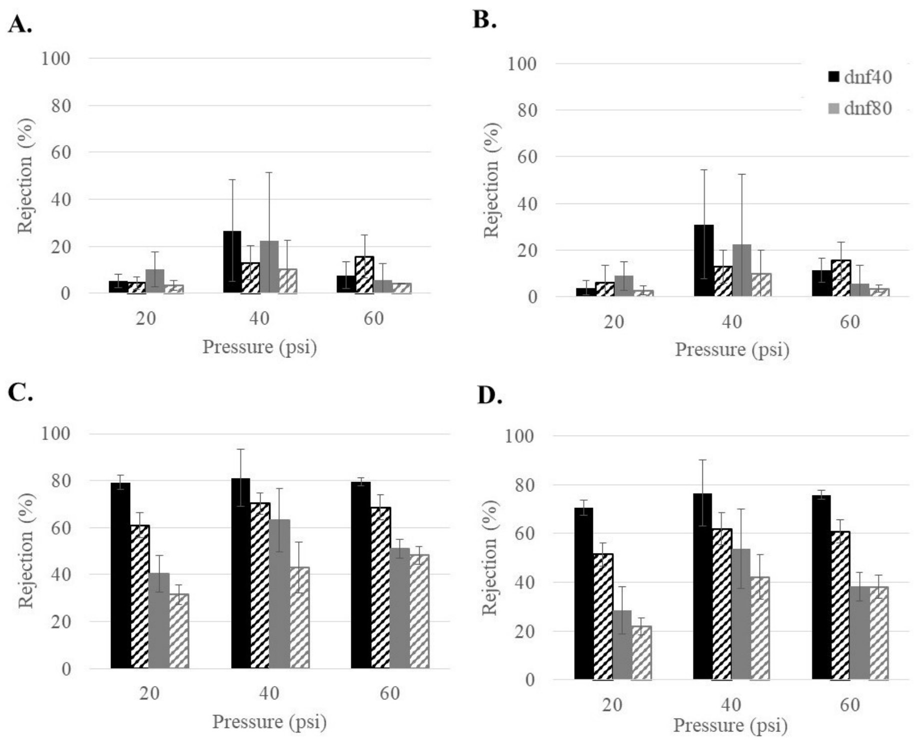

Figure S9. Temperature effects on ion rejection for dnf40 and dnf80 for A. Na^{+}, B. K^{+}, C. Mg^{2+}, and D. Ca^{2+}. Color indicates the membrane (black = dnf40, gray = dnf80) and texture indicates the temperature (solid = room, striped = physiological).
